# Practice–Research Partnerships and Mentoring to Foster Evidence-Based Decision Making

**DOI:** 10.5888/pcd11.140144

**Published:** 2014-05-29

**Authors:** Ross C. Brownson

**Affiliations:** Author Affiliations: Ross C. Brownson, Washington University in St Louis, Missouri.

The uncreative mind can spot wrong answers. It takes a creative mind to spot wrong questions.

— Anthony Jay, British writer and journalist

When practitioners take an evidence-based approach to chronic disease prevention, they undertake several important processes (1): 1) making decisions based on the best available scientific and rigorous program evaluation evidence; 2) applying program planning and quality improvement frameworks; 3) engaging the community and stakeholders in assessment and decision making; 4) adapting evidence-based interventions for specific populations or settings; and 5) conducting sound evaluation. By using an evidence-based approach, activities in public health practice are explicitly linked with the underlying scientific evidence that demonstrates the causes of chronic diseases, epidemiologic patterns of chronic diseases and risk factors, intervention effectiveness, and external validity.

The National Cancer Institute’s Research to Reality (R2R) Mentorship Program has a set of competencies that are closely aligned with this concept of evidence-based public health (EBPH) (2). In keeping with R2R program objectives, the articles in this special collection of *Preventing Chronic Disease* provide some of the best examples to date showing how practice–academic partnerships can produce new scientific information that is timely and relevant for practitioners as they seek to take a more evidence-based approach to chronic disease prevention. This collection builds on recent work from Jacobs and colleagues (3), who presented tools for EBPH practice, and Kaplan and colleagues (4), who showed how these tools can be applied in a state public health agency. An underlying premise for EBPH is that many workers in public health lack formal training in public health sciences (eg, epidemiology, behavioral sciences), making it crucial for individuals and agencies to make a sustained commitment to lifelong learning, a concept exemplified in the principles articulated by R2R.

The systematic approach taken toward mentoring in the R2R program is highly innovative. Its premise is that, for any new field to prosper, both human and intellectual capital must be developed to generate new knowledge and narrow the research-to-practice gap. The R2R program, which has been designed to create a virtual community of practice, fosters a collaborative learning environment that supports many of the goals in the National Cancer Institute’s Strategic Plan (5) and produces science that is relevant for cancer control and for the broad field of public health. The literature on mentoring in the health sciences forms the basis for evidence-informed mentoring (Table). Although the R2R program has not addressed in detail all 8 domains described in the Table, it has successfully addressed many of them, as illustrated in the article by Sanchez and colleagues (6).

**Table:** Domains, Definitions, and Examples of Evidence-Informed Mentoring for Practice-Based Research in Public Health

Domain	Definition	Examples
Skill-building	Providing direct tutelage to help mentees attain competence in specific areas deemed essential to success in research	Writing for publication Conducting experiential learning Building practice partnerships in agencies addressing chronic disease disparities
Sharing resources and infrastructure	Making available any resources that will enhance the mentee’s productivity	Making data available for analysis Providing support for grant proposal preparation Building and participating in communities of practice
Performance feedback	Critically and routinely evaluating mentee’s performance and progress toward goals	Identifying strengths and weaknesses Posing challenging questions Providing recommendations for improvement
Providing opportunity	Creating or sharing opportunities to develop professional skills and generate scholarly dissemination products	Including mentees in developing and reviewing scholarly products Identifying and sharing learning opportunities such as seminars and workshops
Career planning	Helping identify a desired career path and developing a strategic plan to get there	Facilitating the mentee’s identification and refinement of career goals Planning a program of research or practice
Professional networking	Helping mentees make connections with other individuals and organizations in the field	Creating opportunities to interact with established scholars and practitioners Facilitating mentee interaction with other mentees
Professional socialization	Helping mentees understand the field and professional norms and roles within it	Sharing knowledge of the field and professional norms Preparing mentee for new professional experiences such as interviews or grant reviews
Providing emotional support	Understanding and helping mentees cope with stressors and setbacks both related and unrelated to their career or program of study	Listening to mentee’s concerns Providing encouragement and support Facilitating problem-solving

The fruits of practice–academic mentoring and collaboration are shown in this set of articles. Several pieces build on Purcell et al (2) to illustrate the reach and effectiveness of R2R program elements. Others describe macro-level processes that are likely to improve chronic disease prevention (eg, greater use of practice-based evidence, better value from and approaches to effective partnerships). Multiple articles describe specific intervention approaches (eg, adapting interventions, testing effectiveness) designed to address specific diseases or risk factors (eg, colorectal cancer screening, physical inactivity). Building on the themes in these articles, this essay describes several current and future priority issues.

## The Value of and Need for More Practice-Based Evidence

Too often, the evidence available for public health practice is generated and applied through separate, nonoverlapping processes. Researchers, often relying on well-funded and professionally staffed grants, are responsible for generating evidence for effective interventions. This intervention evidence (the “push”) is then handed off to practitioners and policy makers who are charged with implementing a program or policy (the “pull”). As noted by Green, if we are seeking to foster evidence-based practice, we need more practice-based evidence (7) (ie, evidence developed in real world settings via partnerships between practitioners and researchers).

This type of practice-based research is more likely to take into account the important concept of context — the difficult-to-measure characteristics of the agency; the community; and the sociocultural, political, or economic surroundings in which an intervention is to be implemented, adapted, or evaluated (1). Contextual factors have an effect on external validity (ie, the degree to which findings from a study or set of studies can be generalizable to and relevant for populations, settings, and time periods other than those in which the original studies were conducted) (8). Practice-based research satisfies one or more of the following principles: 1) use of participatory research approaches to engage stakeholders in identifying study topics; 2) evaluation of ongoing program and policies (sometimes called natural experiments); 3) placement of greater emphasis on external validity; 4) use of new networks for conducting research (eg, practice-based research networks); and 5) use of systems science methods to understand the complexity and context underlying public health issues. The articles in this issue clearly illustrate the value of practice-based research.

## The Need to Pay Greater Attention to Adaptation and Implementation of Evidence-Based Public Health

A core issue facing dissemination and implementation research involves the concept of how adapting an intervention influences effectiveness. Although the evidence base on effective chronic disease interventions has grown enormously in the past few decades, knowledge about how to adapt, implement, and evaluate interventions is lacking for many settings and populations. A key challenge when adapting an intervention is the tension between fidelity (keeping the key ingredients of an intervention that made it successful) and adaptation (the ability to fit the community or setting of interest). Adapting interventions from one setting to another requires considerations regarding the extent to which the determinants of the issue are comparable (which determines whether the intervention focus is or is not appropriate) and how contextual differences (eg, political environment, health care systems) may affect the intervention. Lee and colleagues developed a useful approach for planned adaptation that includes 4 steps: 1) examining the evidence-based theory of change, 2) identifying population differences, 3) adapting the program content, and 4) adapting evaluation strategies (9).

## The Role of Engagement and Partnerships

Participatory research methods have the potential to address chronic diseases by involving community members and stakeholders in the decision-making processes, thus enhancing the relevance and overall quality of research. Within community and public health settings, participatory research builds trust, respect, capacity, empowerment, accountability, and sustainability (10), all of which are critical to improving the intervention’s chances for success. This process has tremendous potential for testing the effectiveness of interventions and for disseminating EBPH. As illustrated in the articles in this collection, community engagement and participation can play roles across a wide spectrum, ranging from engaging consumers, patients, or practitioners as advisors, to hiring research staff from communities of focus, to full participation from community members in all research activities. In taking these steps, researchers will better recognize the practical application of their scientific findings. The most effective strategies to bridge the gap between research and practice are likely to have at their heart effective partnerships between academicians, practitioners, and policy makers.

## The Need to Understand How to Design for Dissemination

Effective dissemination and implementation of evidence-based interventions is a formidable challenge. In part, this is due to differing priorities. For researchers, the priority is often on discovery (not application) of new knowledge; whereas for practitioners and policy makers, the priority is often on practical ways of applying these discoveries in their settings. Research on how to disseminate evidence-based interventions has now taught us several important lessons: 1) multicomponent, active strategies are often the most effective; 2) leadership matters but is not sufficient; 3) provider behavior is difficult to change and (when changed) is even harder to sustain; and 4) systems are complex and change is recursive. Yet most of these lessons have been learned from studies with early adopters in high-resource settings. We have yet to learn the lessons of how to change public health in more challenging settings. Several articles in this collection take on that challenge.

## Future Research

These articles lead to areas where additional research is warranted, for example —

- How to emphasize building strategic partnerships early in the research process.
- New and more rapid methods for determining when a new program or policy is ready for adoption in a nonresearch setting (eg, exploratory evaluation).
- Ways of ensuring that an intervention is developed in ways that match well with adopters’ needs, assets, and time frames.

Many of these challenges need particular attention in settings with high health-related disparities where system and resource constraints are great and where delivery systems are underdeveloped.

Public health history teaches us that a long latency period often exists between the scientific understanding of a viable disease control method and its widespread application on a population basis. By expanding the evidence base for public health and applying the evidence already in hand, we can shorten the latency period and begin to fully achieve the promise of prevention. In the current era of level or shrinking public health budgets, it is more important than ever to increase the effectiveness and efficiency of public health services by applying the principles of EBPH.
